# Rebamipide with Proton Pump Inhibitors (PPIs) versus PPIs Alone for the Treatment of Endoscopic Submucosal Dissection-Induced Ulcers: A Meta-analysis

**DOI:** 10.1155/2020/7196782

**Published:** 2020-09-28

**Authors:** Junyuan Liu, Zhencheng Xiong, Xuhua Geng, Meihua Cui

**Affiliations:** ^1^Peking University Aerospace School of Clinical Medicine, Beijing 100049, China; ^2^Department of Gastroenterology, Aerospace Center Hospital, Beijing 100049, China; ^3^Institute of Medical Technology, Peking University Health Science Center, Beijing 100191, China; ^4^Peking University Third Hospital, Beijing 100191, China

## Abstract

**Objective:**

To contrast the effect of rebamipide with proton pump inhibitors (PPIs) versus PPIs alone for the treatment of endoscopic submucosal dissection (ESD-) induced ulcers.

**Methods:**

PubMed, Embase, the Cochrane library, the WanFang database, and China National Knowledge Infrastructure (CNKI) were searched to identify studies that met the inclusion criteria.

**Results:**

Nine randomized controlled trials (RCTs) were recognized, including 1170 patients. In general, rebamipide plus PPIs acted better than PPIs alone against ESD-induced ulcers at four weeks (RR = 1.42, 95% CI: 1.13-1.78, *P* = 0.003) but showed no significant differences at eight weeks (RR = 1.03, 95% CI: 0.97-1.10, *P* = 0.315). The use of rebamipide plus PPIs was superior to PPIs alone for ESD-induced ulcers greater than 20 mm in size (20-40 mm: RR = 1.98, 95% CI: 1.22-3.23, *P* = 0.006; >40 mm: RR = 5.14, 95% CI: 1.49-17.74, *P* = 0.010). In addition, rebamipide plus PPI therapy was discovered to be significantly more effective than PPIs alone for lower ESD-induced ulcers (RR = 1.82, 95% CI: 1.04-3.20, *P* = 0.037). There were no significant differences between the treatment groups with the ulcer reduction rate.

**Conclusion:**

Evidences now available show rebamipide plus PPIs is practical for protecting against ESD-induced ulcers at four weeks but not at eight weeks, especially large ulcers (>20 mm). However, we still need more high-quality RCTs in the future to supplement our conclusions.

## 1. Introduction

Endoscopic submucosal dissection (ESD), first evolved in the late 1990s and early 2000s, is presently a diffusely adopted endoscopic resection technique for early gastric cancers (EGC) and some gastric adenomas [1]. ESD is minimally invasive for the patients, and the en bloc rate is higher than the endoscopic mucosal resection, no matter the injury size [2]. However, the use of ESD may lead to deep and large gastrointestinal ulcers and complications such as perforation, bleeding, indigestion, and abdominal pain [3]. Proton pump inhibitors (PPIs) are currently the main drugs for the treatment of peptic ulcer, usually used for ESD-induced ulcer [4]. However, some studies have indicated that the healing effect of PPIs alone is not sufficient for the ESD-induced ulcers within the duration of treatment [5].

Rebamipide, as a mucosal protective drug, can not only increase the production of endogenous prostaglandins but also has the cytoprotective antiulcer effects [6]. The treatment of peptic ulcers by using rebamipide is valid, and rebamipide can reduce the recurrence ratio, without impacting the *Helicobacter pylori* infection condition of the patients in the former researches [7]. Moreover, experimental trials have confirmed the protective effect of rebamipide against non-steroidal anti-inflammatory drugs (NSAIDs-) induced gastrointestinal mucosal lesion [8]. Some studies have shown that the effect of rebamipide plus PPIs is the same as or better than PPIs alone [9]. Therefore, we conducted a meta-analysis to evaluate the efficacy of rebamipide plus PPIs in the treatment of ulcers after ESD compared with PPIs alone.

## 2. Materials and Methods

This meta-analysis was conducted based on the Preferred Reporting Items for Systematic Reviews and Meta-Analyses (PRISMA) statement [10].

### 2.1. Search Strategy

To ascertain all studies comparing the efficacy and safety of rebamipide plus PPIs with PPIs alone for the treatment of ulcers after ESD, we searched on the PubMed, Embase, the Cochrane Library database, China National Knowledge Infrastructure (CNKI), and the WanFang database for all articles published up to June 2020. Search terms included: “Endoscopic submucosal dissection,” “ESD,” “proton pump inhibitors,” “PPIs,” and “rebamipide.” There were no language constraints. Study citations and abstracts were assembled, and full papers were searched to filtrate for possibly relevant literature. The abovementioned process of literature search and selection was independently accomplished by two researchers. All disagreements were resolved by consensus.

### 2.2. Study Selection

The inclusion criteria are as follows: (1) all studies contained the comparison of rebamipide plus PPIs versus PPIs alone for the treatment of ulcers after ESD and (2) the relevant data of the outcome measures of the two groups can be successfully extracted and analyzed.

The exclusion criteria are as follows: (1) non-RCTs, qualitative studies, or studies without withdrawable data; (2) the study population or trial size was not clear; and (3) case reports, editorials, comments, and reviews, or just abstract alone were ruled out.

### 2.3. Data Extraction and Quality Assessment

The withdrawable data were the following: first author, year of publication, country, study type, age, gender, number of participants (rebamipide plus PPIs : PPIs), dose, medication duration, lesion size, tumor location, and endpoints. We used the six-stage Sakita and Fukutomi table (active (A1, A2), healing (H1, H2), and scarring (S1, S2)) to classify the grade of healing of the ulcers [11]. Two researchers independently extracted the data. The divergences in the data extraction process were resolved by discussion.

A quality assessment of each included RCT was executed by two researchers with the Cochrane Handbook for Systematic Reviews [12].

### 2.4. Statistical Analysis

We analyzed the data by using RevMan 5.3 and Stata version 16.0. Different studies compared the efficacy and safety of rebamipide plus PPIs versus PPIs alone for ESD-induced ulcers. We analyzed the results of the duration of treatment, specimen size, location of resection, and reduction rate of ulcers for subgroup analysis. The risk ratio (RR) and 95% confidence interval (CI) was used to analyze dichotomous data, such as the reduction rate and healing rate of different duration of treatment, location of resection, and specimen size. The weighted mean differences (WMD) were used to analyze continuous data. The heterogeneity was investigated by using the *Q* test and the *I*^2^ test. The values of *I*^2^ 25%-50%, 50%-75%, and >75% were considered as low, moderate, and high heterogeneity, respectively [13]. We perform the random-effects model when *I*^2^ > 50% and *P* < 0.1. Otherwise, the fixed-effects model is executed. *P* < 0.05 was regarded as statistically significant in all tests. Begg's test was performed to assess potential publication bias [14].

## 3. Results

### 3.1. Selection of Studies

**Figure 1:**
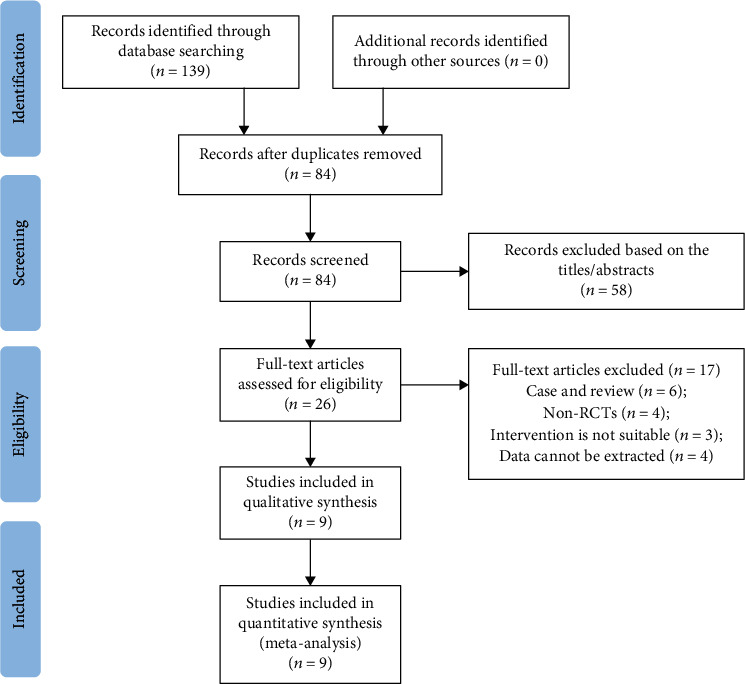
Flowchart of the study selection process.

A sum of 139 records was confirmed by searching with the keywords and free words. After inspecting the titles and abstracts, 113 records were excluded because of duplication or irrelevance; 17 records were excluded as a result of insufficient data from the residual articles by full review. Eventually, 9 RCTs were involved to analyze [9, 11, 15–21].  Figure 1 shows the flow of study inclusion.

### 3.2. Study Characteristics

The characteristics of the nine studies with 1170 patients published between 2010 and 2019 are listed in Table 1. All studies compared the effect of rebamipide plus PPIs versus PPIs alone for the healing of ESD-induced ulcers. All included trials were implemented in Asia (1 in China, 1 in Korea, 7 in Japan). The participants in each study took various types and different doses of PPIs, such as rabeprazole 10 mg/day, omeprazole 20 mg/day, lansoprazole 30 mg/day, and pantoprazole 40 mg/day. The sample size of four trials exceeded 100 [17, 18, 20, 21].

### 3.3. Risk of Bias

**Figure 2:**
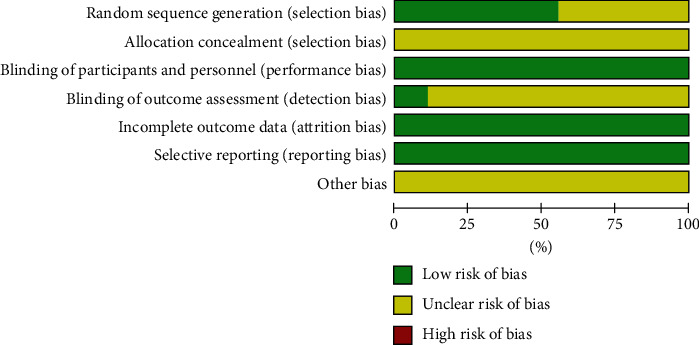
Risk of bias assessment.

**Figure 3:**
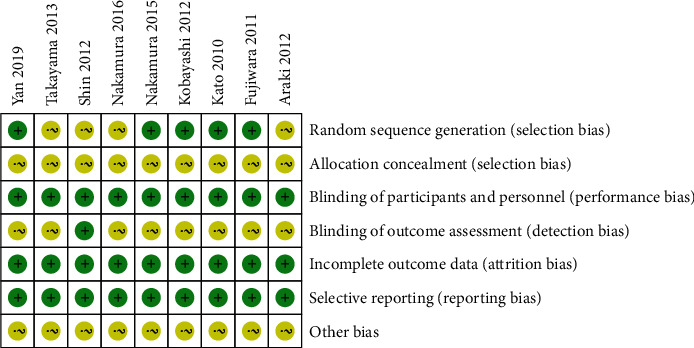
Risk of bias summary: +, low risk of bias; -, high risk of bias; ?, unclear risk of bias.

Random sequence generation was found in five studies [11, 15, 17, 19, 21]. Blinding of participants and personnel was found in nine studies [9, 11, 15–21]. Blinding of outcome assessment was found in one study [18]. The results of the quality assessment in this meta-analysis are shown in Figures 2 and Figure 3.

### 3.4. Outcomes of the Meta-analysis

We conducted this meta-analysis of the included 9 RCTs [9, 11, 15–21]. Among them, the healing rate of different duration of treatment was the primary outcome measure. The specimen size, location of resection, and reduction rate of ulcers were the secondary outcome measures.

#### 3.4.1. Duration of Treatment

**Figure 4:**
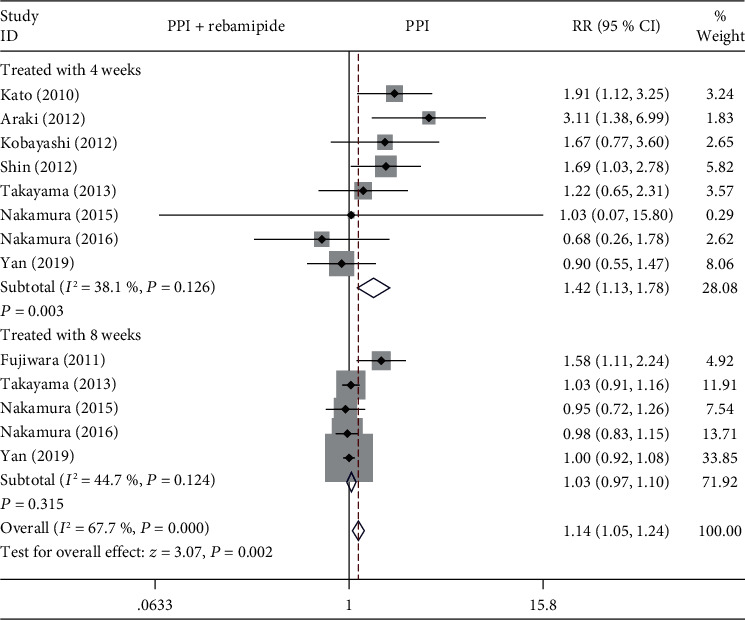
Forest plot showing the effect of rebamipide plus PPIs versus PPIs alone for the healing of ESD-induced ulcers in terms of the duration of treatment.

We performed an analysis to assess the healing effect of rebamipide plus PPI therapy compared with PPIs alone within four or eight weeks. Eight studies reported 4 weeks of treatment outcomes [9, 11, 16–21]. Five studies reported 8 weeks of treatment outcomes [9, 15, 19–21]. As shown in Figure 4, there was a statistically significant difference between the rebamipide plus PPIs group and PPIs alone group after the four weeks treatment for the ESD-induced ulcers (RR = 1.42, 95% CI: 1.13-1.78, *P* = 0.003, *I*^2^ = 38.1%). However, there were no statistically significant differences between the two groups which received eight weeks of treatment (RR = 1.03, 95% CI: 0.97-1.10, *P* = 0.315, *I*^2^ = 44.7%).

#### 3.4.2. Specimen Size

**Figure 5:**
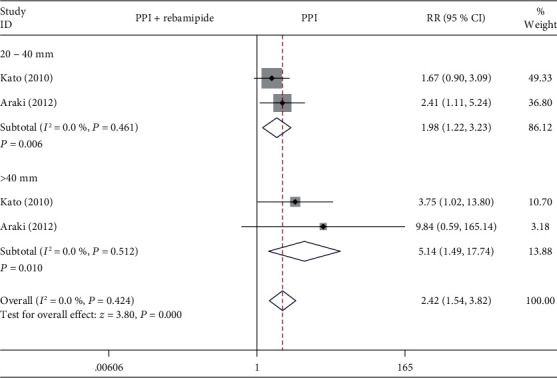
Forest plot showing the effect of rebamipide plus PPIs versus PPIs alone for the healing of ESD-induced ulcers in terms of the specimen size.

In two studies [11, 16], we evaluated the healing effect of rebamipide plus PPI therapy compared with PPIs alone on different specimen sizes (Figure 5). The data indicated a prominently higher value of healing rate of ESD-induced ulcers in the rebamipide plus PPIs group than in the PPIs alone group with the specimen size 20-40 mm (RR = 1.98, 95% CI: 1.22-3.23, *P* = 0.006, *I*^2^ = 0%) and >40 mm (RR = 5.14, 95% CI: 1.49-17.74, *P* = 0.010, *I*^2^ = 0%).

#### 3.4.3. Location of Resection

**Figure 6:**
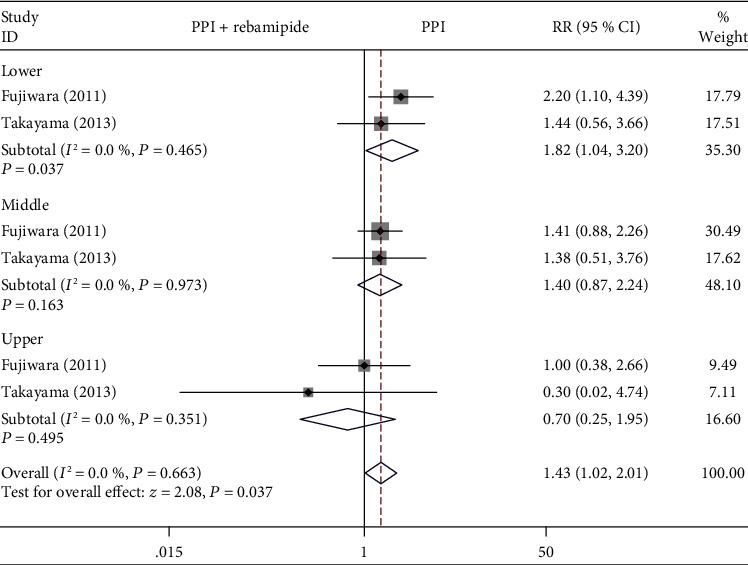
Forest plot showing the effect of rebamipide plus PPIs versus PPIs alone for the healing of ESD-induced ulcers in terms of the specimen location of resection.

As shown in Figure 6, there was a comparison of the healing effect with rebamipide plus PPI therapy versus PPIs alone on different locations of resection (lower, middle, upper stomach) in two studies [9, 15]. There was a statistically significant difference between the two groups in the patients with lower ESD-induced ulcers (RR = 1.82, 95% CI: 1.04-3.20, *P* = 0.037, *I*^2^ = 0%). There were no statistically significant differences on the middle and upper ESD-induced ulcers between the two groups based on the results of the pooled analysis (middle: RR = 1.40, 95% CI: 0.87-2.24, *P* = 0.163, *I*^2^ = 0%; upper: RR = 0.70, 95% CI: 0.25-1.95, *P* = 0.495, *I*^2^ = 0%).

#### 3.4.4. Reduction Rate

**Figure 7:**
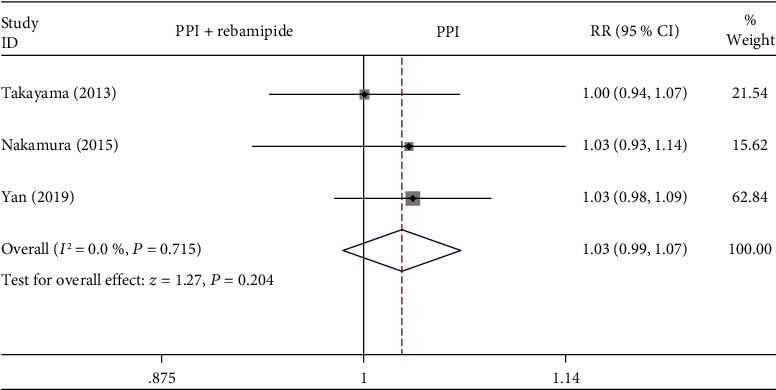
Forest plot showing the effect of rebamipide plus PPIs versus PPIs alone for the healing of ESD-induced ulcers in terms of the reduction rate.

The ulcer reduction rate was assessed between the rebamipide plus PPI therapy group and PPIs alone group in three studies [9, 19, 21]. As shown in Figure 7, there were no statistically significant differences in the ulcer reduction rate between the rebamipide plus PPIs group and PPIs alone group (RR = 1.03, 95% CI: 0.99-1.07, *P* = 0.204, *I*^2^ = 0%).

#### 3.4.5. Adverse Events

Fujiwara et al. [15] reported that one patient underwent bleeding from the iatrogenic ulcers after ESD. Nakamura et al. [19] reported that bleeding occurred in three patients in the rebamipide plus PPIs group. There were no complications involved in the drugs used after ESD in any of the participants.

### 3.5. Publication Bias

**Figure 8:**
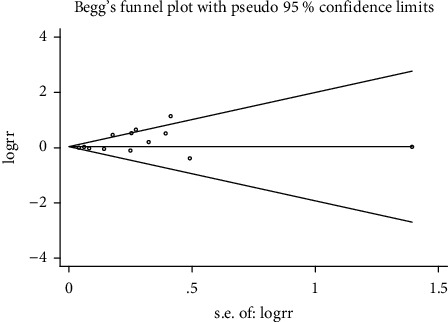
Begg's funnel plot to evaluate publication bias.

Using Begg's funnel plot, the potential publication bias in the included studies was evaluated (Figure 8). No publication bias was detected by Begg's test due to the *P* value >0.05 for the healing effect of rebamipide plus PPI therapy compared with PPIs alone.

### 3.6. Sensitivity Analysis

**Figure 9:**
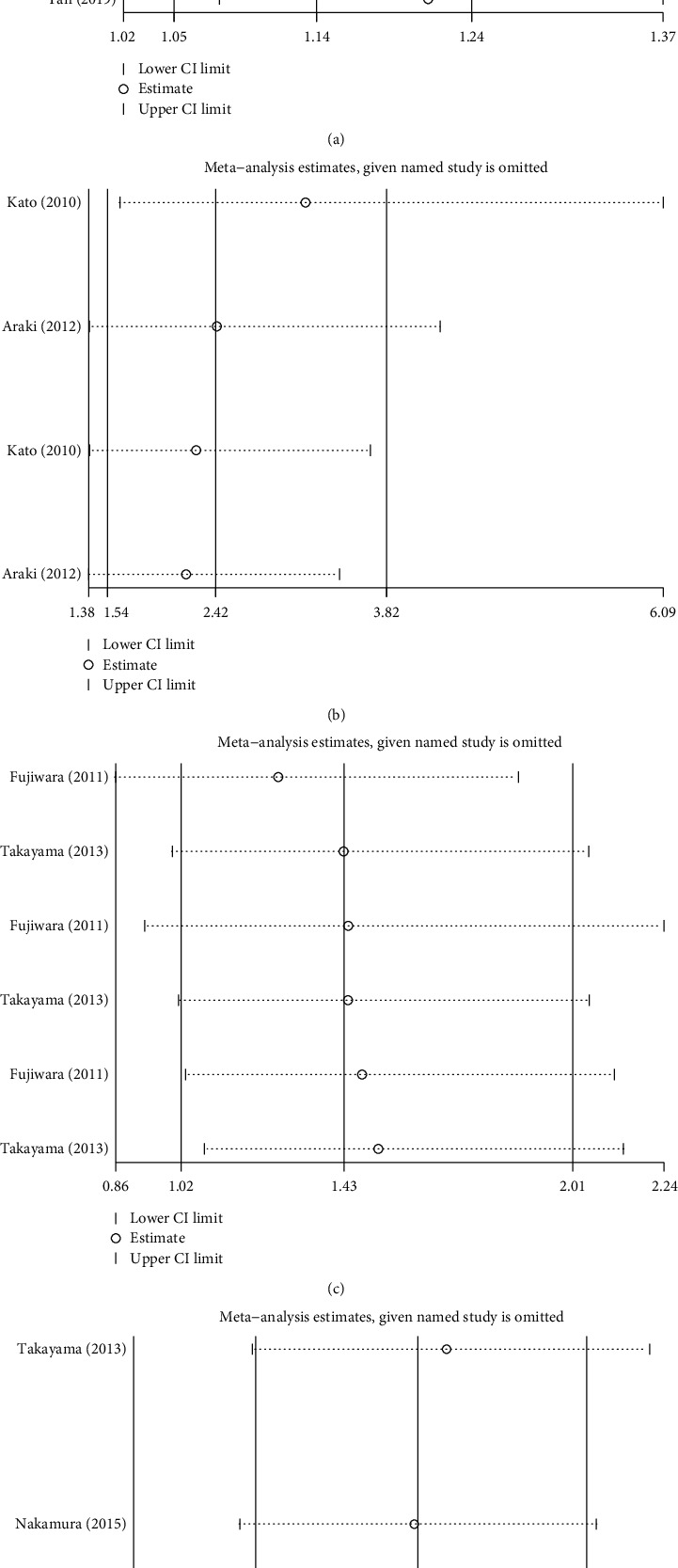
Sensitivity analysis for the confirmation of the stability of the pooled result: (a) duration of treatment, (b) specimen size, (c) location of resection, and (d) reduction rate.

A sensitivity analysis was implemented to value the dependability of this meta-analysis. In eight trials, the duration of treatment was eight weeks for the healing of ESD-induced ulcers, and the duration of the five trials was four weeks. Several sets of sensitivity analysis were used to test the robustness of the pooled analysis results of the outcome measures (Figures 9(a)–9(d)). We found that the results of the meta-analysis did not change after excluding each article.

## 4. Discussion

ESD is the major class of endoscopic resection technique currently used to treat the superficial gastrointestinal neoplasms or lesions, regardless of the size or location. There are larger artificial gastric ulcers or delayed bleeding after the use of ESD. PPIs are the standard treatment for the healing of ulcers after ESD. However, Oh et al. [22] described that the premier ulcer size influenced ulcer healing by PPIs at 4 weeks after ESD, and Kakushima et al. [23] showed that 4 weeks of PPI treatment was not enough for ESD-induced ulcers of large size to heal and that 8 weeks was required. Therefore, it seems that the management of PPIs alone may not be sufficient for the ESD-induced ulcers to heal; the combination therapy is needed. Rebamipide, as a mucosal protective agent, can enhance the production of endogenous prostaglandins, inhibit the reduction of mucosal blood flow, suppress increases in permeability, scavenge free radicals, and has an anti-inflammatory effect [24–27].

We conducted this meta-analysis to explore the effects of treatment with rebamipide plus PPIs versus PPIs alone for the ulcers after ESD. Previous studies indicated that PPIs alone for the ulcers after ESD was not sufficient [11]. In this meta-analysis, the results showed that rebamipide plus PPIs is superior to PPIs alone for healing the ESD-induced ulcers at four weeks; however, there were no significant differences between the rebamipide plus PPIs and PPIs alone with a treatment of eight weeks. In general, the healing rate in the combination therapy group was higher than that in the PPIs alone group. Some studies reported that the healing of ESD-induced ulcers was associated with both the location of resection and the specimen size [11]. In this meta-analysis, we evaluated the degree of ulcer healing with respect to the location of resection and specimen size, and we detected significantly higher healing rate after ESD in the rebamipide plus PPIs group than in the PPIs alone group with the specimen size 20-40 mm, particularly with the specimen size >40 mm. We also found a significantly higher healing rate of ulcers after ESD in the rebamipide plus PPIs group than in the PPIs alone group with lower location of resection. Three studies evaluated the reduction rate of ESD-induced ulcers, but there were no significant differences between the treatment groups. In addition, serious complications connected with the drugs used after post-ESD were not noticed in the two groups.

### 4.1. Limitations

Although increasing studies recommend rebamipide as a valid mucosal protective agent, the current data are not plenty for strictly confirming the effectiveness of rebamipide. Therefore, this meta-analysis has some limitations to be addressed. First, the number of inclusive studies was quite limited, and the quality of the containing RCTs was relatively low. Second, all the included studies were in Asia; the racial differences might lead to different reactions to drugs. Third, the various types and different use methods of PPIs might produce a bias. Fourth, some studies lack extractable data, random sequence generation methods, double-blind or triple-blind details, and uniform follow-up time.

## 5. Conclusion

In general, this meta-analysis has presented the crucial profit of rebamipide plus PPIs in managing ESD-induced ulcers. The results of the pooled analysis mainly show that rebamipide plus PPIs is practical for protecting against ESD-induced ulcers at four weeks but not at eight weeks, especially large ulcers (>20 mm). However, more studies with designs of large scale and high quality are still required to further determine the efficacy of rebamipide plus PPIs.

## Figures and Tables

**Table 1:** Characteristics of the included trials.

Authors	Year	Country	Mean age (years) E:C	No. of patients E:C	Male E:C	Interventions E:C		Duration (weeks)
Kato et al. [11]	2010	Japan	73/73	31/31	20/24	PPIs + 100 mg rebamipide 3 times/day	Rabeprazole 10 mg/day	4
Fujiwara et al. [15]	2011	Japan	68/69	30/31	21/24	PPIs + rebamipide 300 mg/day	Omeprazole 20 mg/day	8
Araki et al. [16]	2012	Japan	71/69.5	45/42	30/30	PPIs + 100 mg rebamipide 3 times/day	Omeprazole 20 mg/day, rabeprazole 10 mg/day, or lansoprazole 30 mg/day	4
Kobayashi et al. [17]	2012	Japan	70.0/70.8	85/85	66/68	PPIs + rebamipide 300 mg/day	Omeprazole 20 mg/day or lansoprazole 30 mg/day	4–6
Shin et al. [18]	2012	Korea	63.4/64.9	126/129	101/98	PPIs + 100 mg rebamipide 3 times/day	Pantoprazole 40 mg/day	4
Takayama et al. [9]	2013	Japan	67/70	45/44	31/36	Lansoprazole 30 mg/day, 5 d; then rebamipide 300 mg/day	Lansoprazole 30 mg/day	8
Nakamura et al. [19]	2015	Japan	68/67	33/34	27/28	PPIs + rebamipide 300 mg/day	Rabeprazole 20 mg/day	8
Nakamura et al. [20]	2016	Japan	68.7/70.3	54/55	NP	PPIs + 100 mg rebamipide 3 times/day	Rabeprazole 10 mg/day	8
Yan et al. [21]	2019	China	59.80/59.95	137/133	103/106	PPIs + 100 mg rebamipide 3 times/day	Lansoprazole 30 mg/day + placebo 3 times/day	8

PPIs: proton pump inhibitors; E: treatment group with rebamipide; C: treatment group without rebamipide; NP: not provided.

## Data Availability

The data supporting this meta-analysis is from previously reported studies and datasets, which have been cited.
